# Supplementary material for: Morphological and Chemical Factors Related to Western Flower Thrips Resistance in the Ornamental Gladiolus
Source: Plants (Basel). 2021 Jul 6;10(7):1384. doi: 10.3390/plants10071384 (PMC8309351; doi:10.3390/plants10071384)
Supplement: Supplementary file 1 [file plants-10-01384-s001.zip › plants-1185447-supplementary/Supplementary figures_revised.pdf]

Supplementary Materials

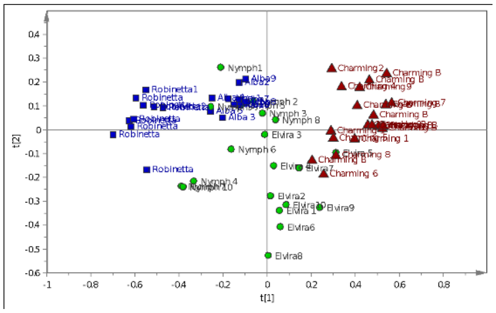

Figure S1. PCA score plot for 14 varieties based on thrips resistance of the varieties. (▲) susceptible varieties, (●) medium varieties and (■) resistant.

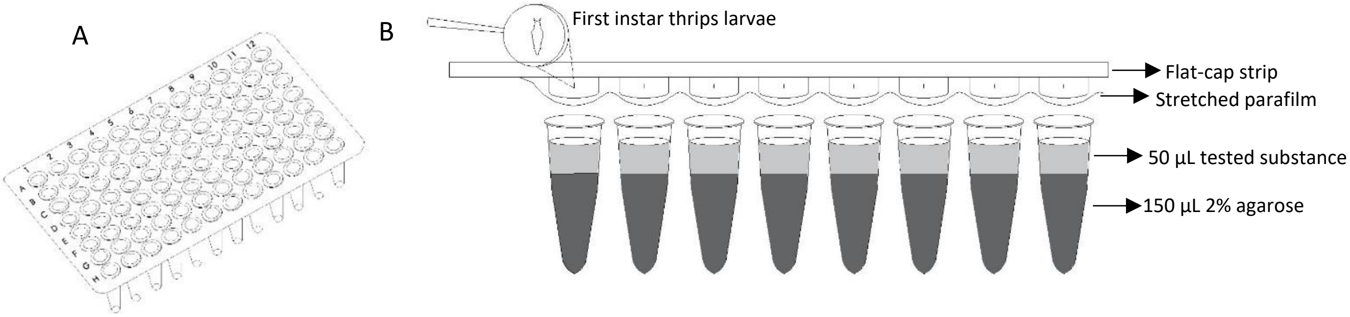

Figure S2. The *in-vitro* thrips bioassay; 96-well plates at top view (A) and at side view (B).
